# 

**Published:** 1861-07

**Authors:** 


AMERICAN.

Another Letter to a Young Physician; to which are appended some other Medical Papers. By James Jackson, M.D., Professor Emeritus of the Theory and Practice of Medicine in Harvard University. 12mo., pp. 179. Boston, 1861.

Microscopical Anatomy of the Lumbar Enlargement of the Spinal Cord. By John Dean, M.D. 4to., pp. 21, with four plates. Cambridge, 1861.

A Manual of Military Surgery; or Hints on the Emergencies of Field, Camp, and Hospital Practice. By S. D. Gross, M.D. 18mo., pp. 186. Philadelphia: J. B. Lippincott & Co., 1861.

A Practical Treatise on Military Surgery. By Frank Hastings Hamilton, M.D. 8vo., pp. 234. New York: Baillière Brothers, 1861.

A Hand-book of Military Surgery. By Charles S. Tripler, M.D., Surgeon United States Army, and George C. Blackman, M.D., Professor of Surgery in the Medical College of Ohio. 12mo., pp. 121. Cincinnati, 1861.

The Classification, Diagnosis, and Prognosis of Tumors. By Theodore Bilbroth, M.D., Professor of Surgery in the University of Zurich. Translated from the German by G. Baumgarten, M.D. 8vo., pp. 46. St. Louis, 1861.

The Modus Operandi of various kinds of Baths; Sea-bathing, Hot and Cold, Physiologically Explained. By John O'Reilly, M.D. 8vo., pp. 23. New York, 1861.

A Treatise on the Practice of Medicine. By Edwin Madson, M.D. 8vo., pp. 705. Philadelphia: Lindsay & Blakiston, 1861.

A Report on Epidemics and Endemics. By Dr. O. C. Gibbs, of Frewsburg, New York. (Reprint.) pp. 24. Philadelphia.

FOREIGN.

On the Treatment of Stricture of the Urethra. By Robert Wade. 12mo., pp. 108. London, 1861.

Transactions of the Obstetrical Society of London. Vol. ii. London, 1861.

On Diseases and Deformities of the Spine, Chest, and Limbs. Part i. By R. Hughes, Surgeon to the Brighton Orthopœdic Hospital. pp. 40. Churchill, London.

Principles of Forensic Medicine. By W. Guy, M.B. Cantab. 2d edition. pp. 534. London: Renshaw, 1861.

A Manual of Qualitative Analysis. By R. Galloway, F.C.S. 3d edition. pp. 246. London, Churchill.

The Anatomist's Vade-Mecum: a System of Human Anatomy. By Erasmus Wilson, F.R.S. pp. 732. 8th edition. London: Churchill, 1861.

Handbuch d. Spec. Path. u. Therapie. Von R. Virchow. Band iii., Lief. 1.

Hints on Insanity. By J. Millar, L.R.C.P. Edin., Medical Superintendent Bothwell House Asylum. pp. 108. Renshaw, 1861.

The Genetic Cycle in Organic Nature; or the Succession of Forms in the Propagation of Plants and Animals. By G. Ogilvie, M.D., Regius Professor of Institutes of Medicine in University of Aberdeen. pp. 296. Aberdeen; Edinburgh; London, 1861.

Clinical Surgery. Diseases and Injuries of the Nose, Larynx, Organs of Circulation, etc. By T. Bryant, F.R.C.S., Assistant Surgeon at Guy's Hospital. Part iii. pp. 151. London, Churchill.

The Forms, Complications, Causes, Prevention, and Treatment of Consumption and Bronchitis; comprising also the Cause and Prevention of Scrofula. By J. Copland, MD., F.R.S. pp. 940. London, Longman & Co.

Practical Observations on the Diseases of the Joints involving Anchylosis, etc. By Bernard E. Brodhurst, Assistant Surgeon to the Royal Orthopœdic Hospital. pp. 120. London, Churchill.

Sore Throat and the Laryngoscope. By M. Prosser James, M.D., Senior Physician to the Metropolitan Dispensary. pp. 155. London, Churchill.

Constipated Bowels; the various Causes and Rational Means of Cure. By S. B. Birch, M.D. pp. 191. London, Churchill.

Communication à l'Académie des Sciences de Paris, sur l'Etablissement de l'Abendberg. By Dr. Guggenbuhl. (Reprint from the Comptes Rendus.) 1860.

Fownes's Manual of Elementary Chemistry. 8th edition. Edited by Dr. H. Bence Jones and Professor Hoffman. pp. 771. Churchill, 1861.

Diphtheria; its Symptoms and Treatment. By W. Jenner, M.D., Special Professor of Clinical Medicine, and Physician to University College, etc. pp. 107. London: Walton & Maberly, 1861.

The Eastern or Turkish Bath; its History, Revival in Britain, etc. By Erasmus Wilson, F.R.S. pp. 167. London: Churchill, 1861.

Ten Lectures Introductory to the Study of Fever. By A. Anderson, M.D., Lecturer on Medicine, Glasgow. pp. 180. London: Churchill, 1861.

